# Influence of Fabrication Processes and Annealing Treatment on the Minority Carrier Lifetime of Silicon Nanowire Films

**DOI:** 10.1186/s11671-017-2006-z

**Published:** 2017-03-31

**Authors:** Shinya Kato, Tatsuya Yamazaki, Yasuyoshi Kurokawa, Shinsuke Miyajima, Makoto Konagai

**Affiliations:** 1 Department of Electrical and Mechanical Engineering, Nagoya Institute of Technology, Showa-ku, Nagoya-shi, Aichi 466-8555 Japan; 2 Department of Physical Electronics, Tokyo Institute of Technology, Meguro-ku, Tokyo, 152-8552 Japan; 3 Graduate School of Engineering, Nagoya University, Nagoya, Aichi 464-8603 Japan; 4 Department of Electrical and Electronic Engineering, Tokyo Institute of Technology, Meguro-ku, Tokyo, 152-8552 Japan; 5 Advanced Research laboratories, Tokyo City University, Setagaya-ku, Tokyo, 158-0082 Japan; 6 FUTURE-PV Innovation, Japan Science and Technology Agency (JST), Koriyama, Fukushima 963-0215 Japan

**Keywords:** Silicon nanowire, Passivation, Minority carrier lifetime

## Abstract

Surface passivation and bulk carrier lifetime of silicon nanowires (SiNWs) are essential for their application in solar cell devices. The effective minority carrier lifetime of a semiconductor material is influenced by both its surface passivation and bulk carrier lifetime. We found that the effective carrier lifetime of SiNWs passivated with aluminum oxide (Al_2_O_3_) was significantly influenced by the fabrication process of SiNWs. We could not measure the effective lifetime of SiNWs fabricated by thermal annealing of amorphous silicon nanowires. Nevertheless, the SiNWs fabricated by metal-assisted chemical etching of polycrystalline silicon displayed an effective lifetime of 2.86 μs. Thermal annealing of SiNWs at 400 °C in a forming gas improved the effective carrier lifetime from 2.86 to 15.9 μs because of the improvement in surface passivation at the interface between the SiNWs and Al_2_O_3_ layers.

## Background

Silicon nanowires (SiNWs) possess promising qualities for use in thin-film solar cells, i.e., they exhibit a strong optical confinement effect that is essential for light trapping in solar cells [1–5]. SiNWs can be fabricated by metal-assisted chemical etching (MAE), which is a low-cost chemical solution-based process and, therefore, presents a potential for low-cost fabrication of SiNW-based solar cells [6–11]. Carrier recombination can significantly affect the performance of solar cells. Thus, the application of SiNWs in solar cells will require the carrier recombination in SiNWs to be examined. One of the most important measures of carrier recombination is the effective carrier lifetime (*τ*_eff_). The value of *τ*_eff_ is very sensitive to the surface recombination and bulk lifetime, and therefore, *τ*_eff_ measurements provide information about surface recombination and bulk lifetime at the same time [12–14]. Owing to the difficulties with the fabrication of isolated SiNWs, however, only few studies examining the *τ*_eff_ of SiNWs have been reported in the literature to date [15, 16]. SiNWs are often fabricated by chemical etching of a crystalline Si (c-Si) substrate [6, 17]. In SiNWs fabricated using the proposed method, the c-Si substrate remains underneath the SiNWs and influences the outcome of *τ*_eff_ measurements. For this reason, fabrication techniques that do not employ the c-Si wafer are needed to remove the influence of the c-Si substrate on the *τ*_eff_ measurements of SiNWs.

In our previous work, isolated SiNWs were fabricated from polycrystalline Si (poly-Si) on a quartz substrate [7–9]. Additionally, our previous work also demonstrated that *τ*_eff_ measurements of isolated SiNWs and their optical characterization are possible [15].

**Fig. 1:**
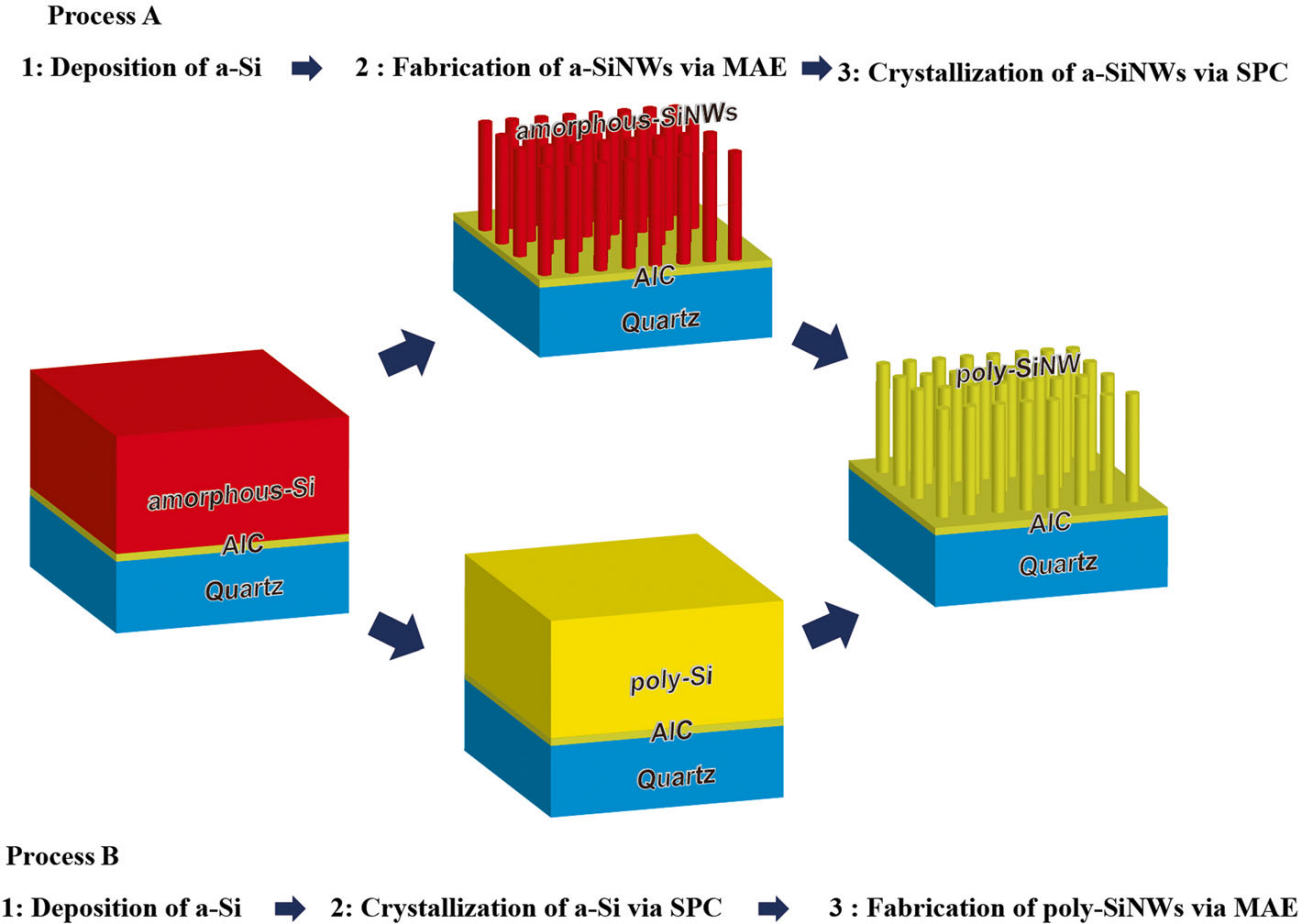
Two different fabrication processes of a poly-SiNW

In this study, we investigated the influence of the fabrication process of isolated SiNWs passivated with aluminum oxide (Al_2_O_3_) on *τ*_eff_. Isolated SiNWs can be fabricated using two different processes, as shown in Fig. 1. Here, SiNWs were fabricated by thermal annealing of amorphous silicon (a-Si) nanowires, marked in Fig. 1 as process A. The a-Si nanowires were fabricated using the MAE process. Additionally, SiNWs were fabricated by MAE of poly-Si, in a process marked as B in Fig. 1. In this case, the poly-Si layer was fabricated by thermal annealing of an a-Si layer. Finally, the influence of annealing time and temperature on the value of *τ*_eff_ was investigated.

## Methods

Isolated SiNWs were fabricated using two different procedures as shown in Fig. 1. A highly doped thin poly-Si layer of thickness 163 nm was fabricated on a quartz substrate by aluminum-induced crystallization (AIC). The details of the AIC process have been reported previously [8]. The AIC-poly-Si layer acts as a seed layer for the crystallization of SiNWs. Subsequently, an intrinsic a-Si (i-a-Si) layer was deposited on the AIC-poly-Si by radio frequency sputtering. In process A, amorphous SiNWs were fabricated by MAE of the i-a-Si layer. During the MAE process, silver particles were deposited on the i-a-Si layer by electroless silver plating. The sample containing silver nanoparticles was immersed in an etching solution comprising HF and H_2_O_2_. Following the formation of amorphous SiNWs, the sample was dipped in an HNO_3_ solution to remove the silver nanoparticles. Finally, the oxide layer formed on the surface of the amorphous SiNWs was removed using an HF solution. The amorphous SiNWs were crystallized by thermal annealing at 800, 900, and 1000 °C for 30 min in a forming gas (FG) under ambient conditions. In process B, the i-a-Si layer was crystallized by solid phase crystallization (SPC) to give the poly-Si prior to nanowire formation through thermal annealing at 900 °C for 30 min in a forming gas at ambient conditions. Finally, the SiNWs were fabricated from the poly-Si layer by MAE.

**Fig. 2:**
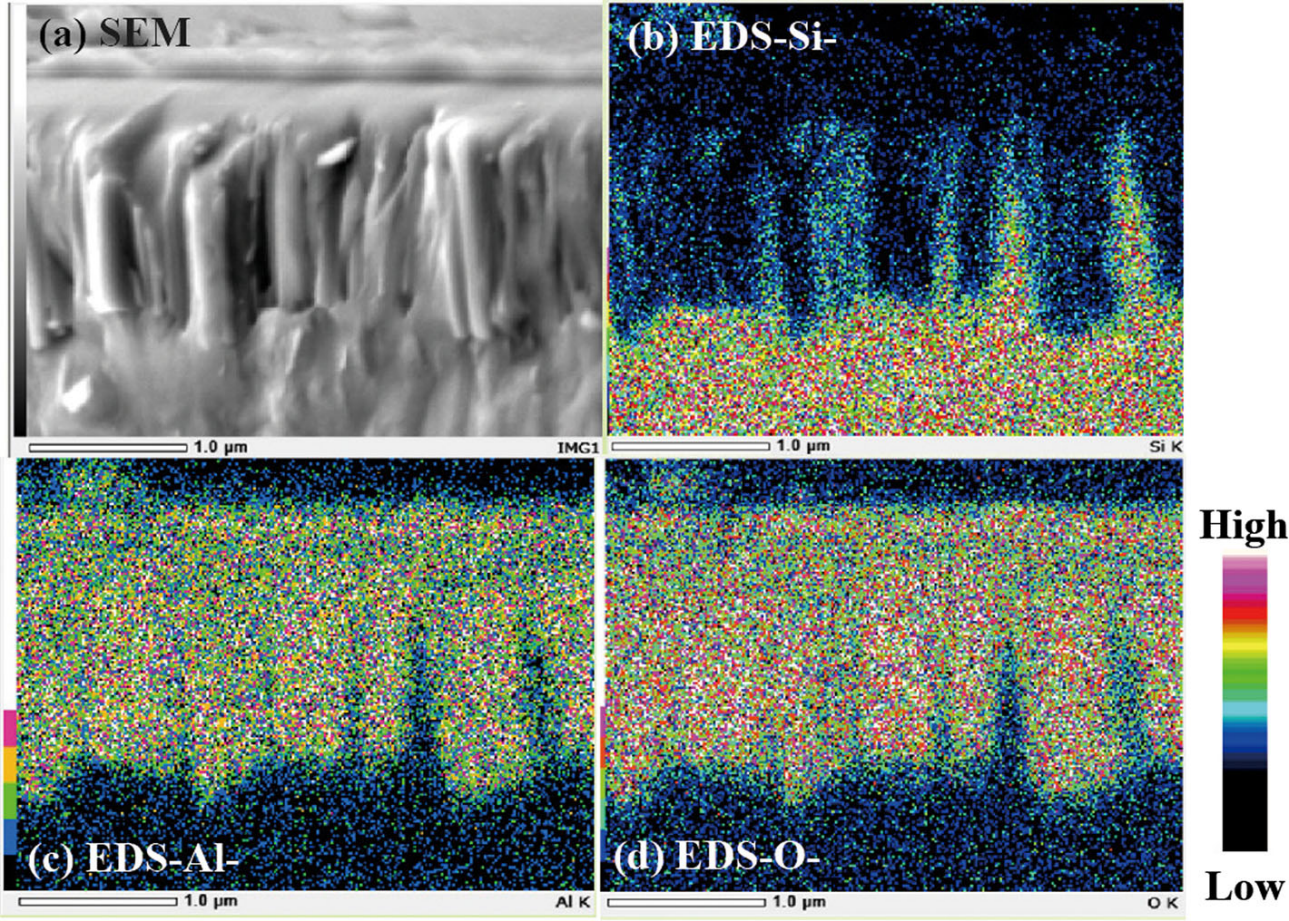
Cross-sectional SEM and EDS images of a-SiNW sample after deposition of Al_2_O_3_. **a** SEM image. **b** EDS image for Si. **c** EDS image for Al. **d** EDS image for O

Following the preparation of SiNWs, an Al_2_O_3_ layer was deposited on the SiNWs for surface passivation by atomic layer deposition. As reported in [6], the effective lifetime of SiNWs lacking a passivation layer cannot be measured because of the extremely high effective surface recombination velocity. Figure 2 depicts the cross-sectional image obtained by using a scanning electron microscope (SEM) and the energy dispersive X-ray spectroscopy (EDS) images of SiNWs containing an Al_2_O_3_ layer. The EDS images clearly showed that the signals arising from Al and O are observed uniformly across the entire area of the sample. This result indicates that the fabricated SiNWs were entirely covered with the Al_2_O_3_ layer. Thermal annealing treatment was conducted in a forming gas (4% H_2_ in N_2_) at 300, 400, and 500 °C. The influence of annealing time on the passivation quality was also investigated.

The structure of the fabricated SiNW samples was characterized by field-emission SEM (FE-SEM) and EDS (JEOL, JSM-7001F). The crystallization of SiNWs was confirmed by Raman spectroscopy (JASCO, NRS-1000, excitation wavelength of 532 nm). The effective carrier lifetime of the samples was measured using a microwave photoconductivity decay (MWPCD) technique and a 904-nm laser (KOBELCO, LTE-1510EP). The interface-state density (*D*_it_) and fixed-charge density (*Q*_ss_) were measured using a noncontact corona-voltage (C-V) technique (SEMILAB Co. Ltd., PV-2000).

## Results and Discussion

**Fig. 3:**
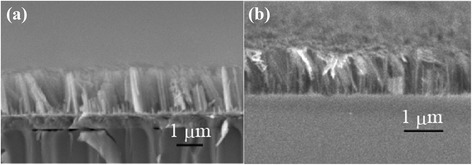
Cross-sectional SEM image of the SiNWs. **a** Process A. **b** Process B

**Fig. 4:**
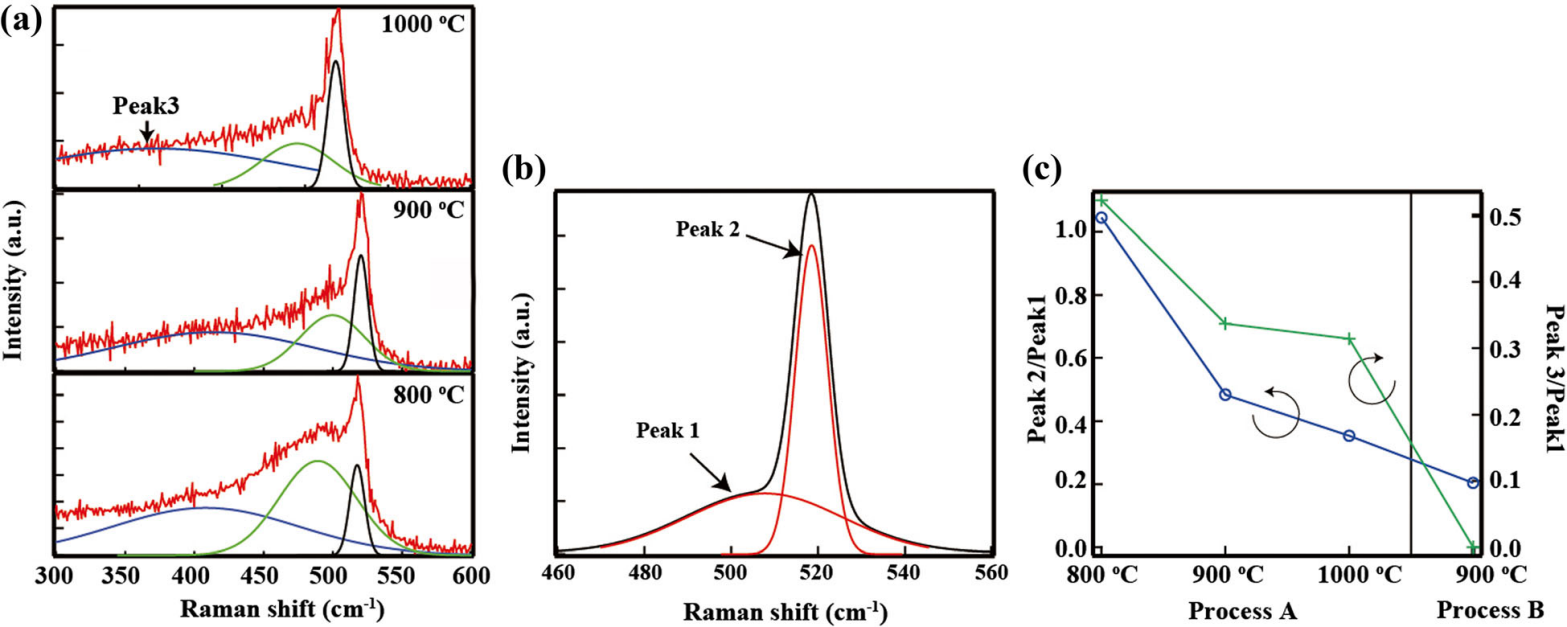
**a** Raman spectra of SiNW fabricated by process A at 800, 900, and 1000 °C. **b** Raman spectra of SiNW fabricated by process B at 900 °C. **c** The ratio of Peak 2/Peak 1 and Peak 3/Peak 1

Figure 3 depicts the cross-sectional SEM images of SiNWs fabricated using process A and process B. The shape of the wires was found to be very similar for both processes, indicating that similar patterns of silver nanoparticles were formed on both the i-a-Si and poly-Si layers. This result demonstrated that the Ag pattern does not depend on the employed fabrication process. In the next step, the internal structure of SiNWs fabricated using different procedures was investigated by Raman spectroscopy. Figure 4a depicts the Raman spectra of SiNWs fabricated using process A. The Raman spectra of SiNWs crystallized at 800, 900, and 1000 °C are shown in Fig. 4a. Three peaks, attributed to crystalline silicon (peak 1), nanocrystalline silicon (peak 2), and amorphous silicon (peak 3), were observed in all spectra. In contrast, only peaks 1 and 2 were observed in the spectra of SiNWs fabricated using process B (Fig. 4b). These results indicate that the SiNWs fabricated using process A contained a residual a-Si phase. The results of Raman spectroscopy, therefore, suggest that the crystallization temperature of a-SiNWs is higher than that of a-Si. This temperature difference can be explained by the Helmholtz free energy of poly-Si. When a polycrystalline silicon with a radius *r* is prepared, the change in the Helmholtz free energy can be calculated as shown in Eq. (1).

1 $$ \varDelta F=-\frac{4}{3}\pi {r}^3\varDelta {F}_v+4\pi \sigma {r}^2 $$

where Δ*F*_ν_ is the variation in Helmholtz free energy between a-Si and poly-Si per unit volume and *σ* is the interface energy. The first term represents the variation in free energy because of phase transformation from a-Si to poly-Si, and therefore, Δ*F* is expected to become negative as the poly-Si grows. The second term represents the increase in the surface area of a-Si that results in the free energy increase. When the influence of the second term is stronger than that of the first term, the a-Si does not crystallize. The crystal nucleation in a-SiNW is affected easily by the nanowire surface, and therefore, the second term is larger than the first term. As a result, the crystallization temperature of a-SiNW is higher compared with that of a-Si. To date, a number of studies reported in the literature have shown that the crystallization temperature of a-Si nanostructure increases [18–20].

In addition to structural characterization, we investigated the *τ*_eff_ of SiNWs/Al_2_O_3_ fabricated using different procedures. The results showed that the *τ*_eff_ of SiNWs/Al_2_O_3_ fabricated using process A could not be measured, whereas that of SiNWs/Al_2_O_3_ fabricated using process B was successfully measured. Most likely, this difference can be attributed to the bulk lifetime of SiNWs since the surface passivation quality of SiNWs fabricated using both processes was essentially identical. The results of Raman spectroscopy confirmed the presence of a residual a-Si phase in the SiNWs fabricated using process A, and this presence significantly affects the bulk lifetime of SiNWs. Overall, these results indicate that process B is more useful for solar cell applications than process A.

**Fig. 5:**
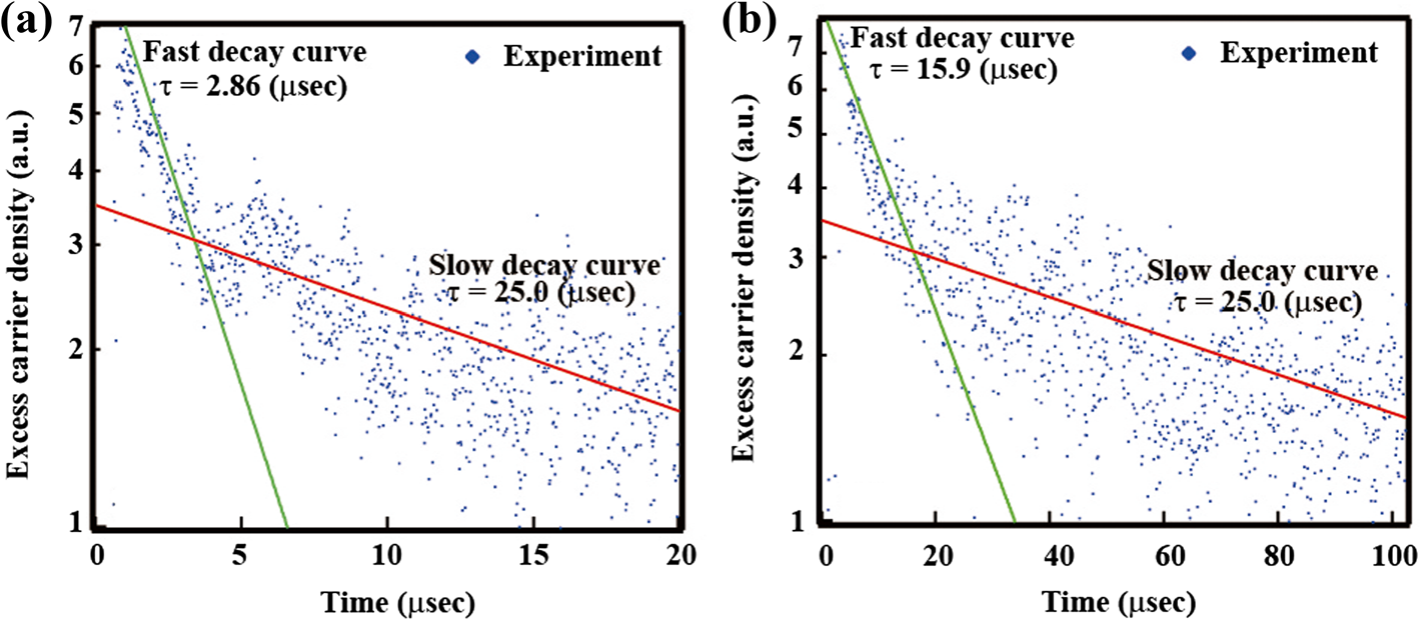
Decay curve of MWPCD signal of SiNW/Al_2_O_3_ sample **a** before and **b** after thermal annealing

The surface passivation quality of the c-Si/Al_2_O_3_ interface is known to be strongly influenced by thermal annealing. Therefore, we investigated the effect of thermal annealing on the *τ*_eff_ of SiNWs/Al_2_O_3_ fabricated using process B. Figure 5 depicts the decay curves of the MWPCD signal of as-deposited and annealed SiNWs/Al_2_O_3_. Each decay curve can be divided into fast and slow decay components. Although the origin of the slow decay has not been clarified, it is most likely related to the carrier trap in SiNWs. We estimated the *τ*_eff_ of as-deposited SiNWs/Al_2_O_3_ from the fast decay curve as 2.86 μs. Thermal annealing of SiNWs/Al_2_O_3_ at 400 °C for 30 min in the forming gas increased the *τ*_eff_ drastically from 2.86 to 15.9 μs.

**Fig. 6:**
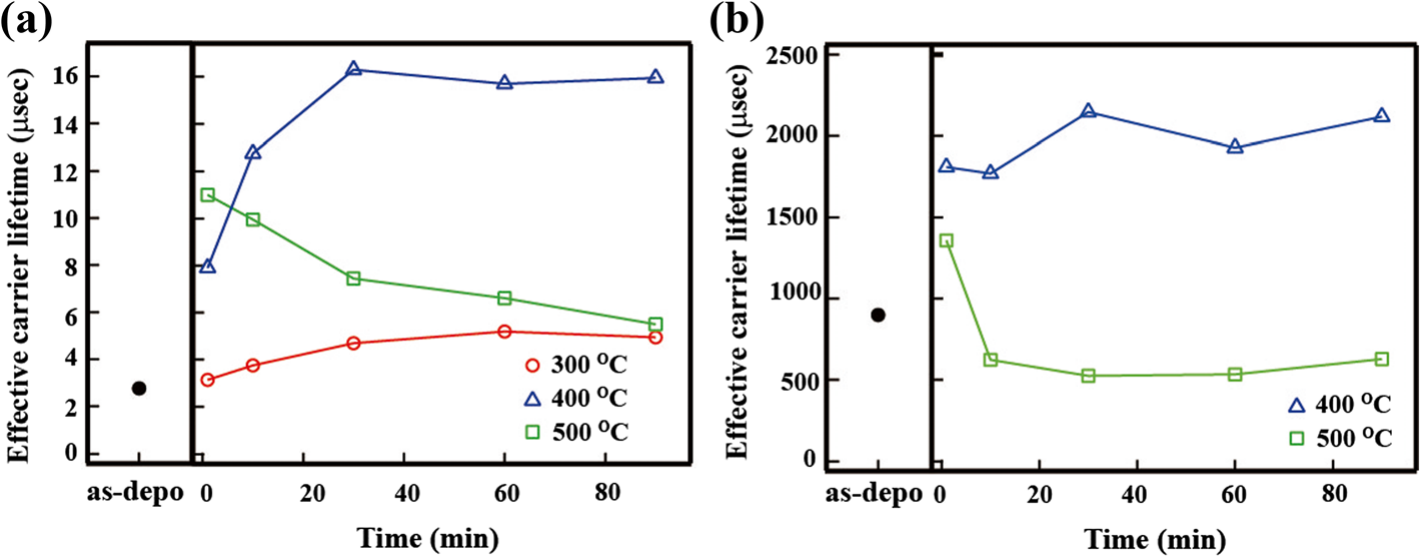
**a** Dependence of *τ*_eff_ of SiNWs/Al_2_O_3_ on annealing time and temperature. **b** Dependence of *τ*_eff_ of c-Si/Al_2_O_3_ on annealing time and temperature

**Fig. 7:**
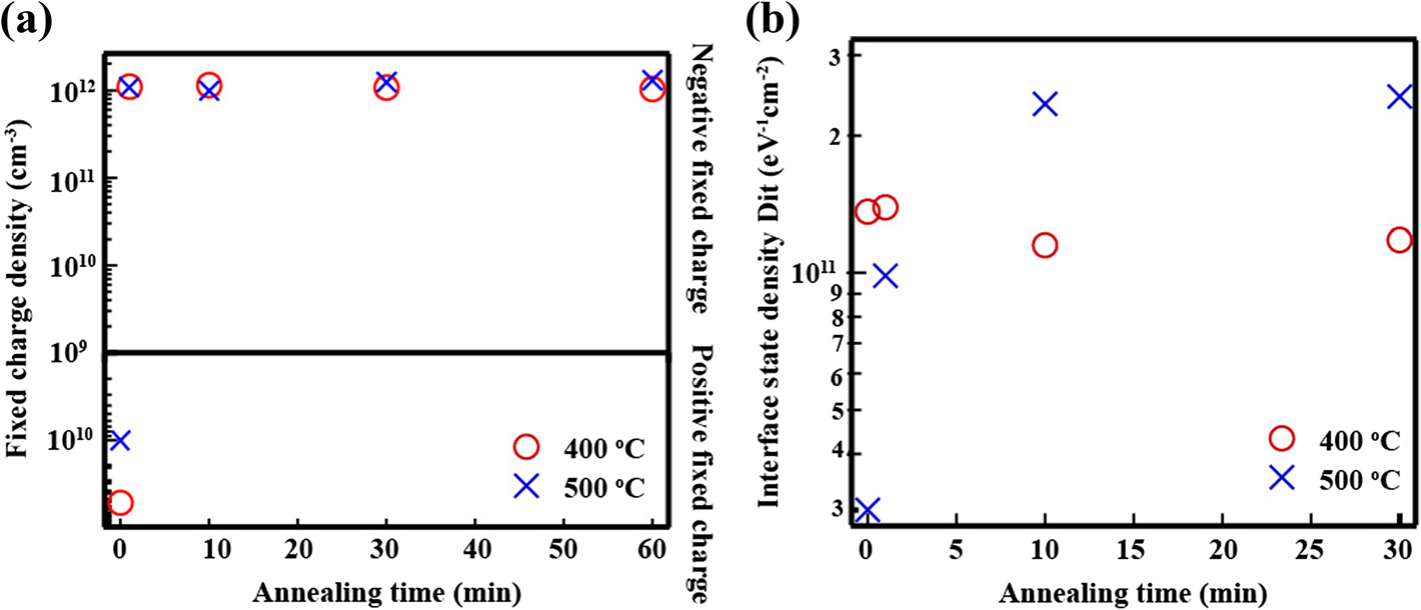
**a** Fixed-charge density (*Q*_ss_) and **b** interface-state density (*D*_it_) as a function of annealing time for different annealing temperature (400 and 500 °C)

Generally, the surface recombination velocity of the Si and Al_2_O_3_ interface decreases during thermal annealing because of the formation of a negative fixed charge [21–24]. Therefore, we examined the effect of thermal annealing on the SiNW/Al_2_O_3_ samples in more detail. Figure 6a depicts the variation in *τ*_eff_ observed as a function of annealing time and temperature. For annealing temperatures of 300 and 400 °C, the *τ*_eff_ increased as the annealing time was increased to 30 min, followed by a region of saturation. Interestingly, the behavior exhibited by *τ*_eff_ at the annealing temperature of 500 °C was very different from that exhibited by *τ*_eff_ at other temperatures. The annealing at 500 °C for 1 min increased the *τ*_eff_ from 3 to 11 μs. However, further increase in annealing time resulted in a monotonic decrease in *τ*_eff_. The difference in behavior observed at the low annealing temperatures (300 and 400 °C) and the high temperature (500 °C) can be attributed to the changes in surface passivation. Figure 6b depicts the changes in *τ*_eff_ of c-Si/Al_2_O_3_ determined as a function of annealing time and temperature. This experiment was performed to examine the influence of annealing on the quality of surface passivation. To eliminate the influence of bulk lifetime, we employed a monocrystalline Si substrate (p-type, [100], 8–12 Ωcm, thickness of 550 μm). The results showed that annealing at 400 °C increased the *τ*_eff_ monotonically, whereas annealing at 500 °C decreased the *τ*_eff_ after a long annealing time. Overall, the behavior observed for the *τ*_eff_ of c-Si/Al_2_O_3_ was similar to that determined for SiNWs/Al_2_O_3_. The *τ*_eff_ of c-Si/Al_2_O_3_ samples is known to be determined by *D*_it_ and *Q*_ss_. Nevertheless, determining *D*_it_ and *Q*_ss_ at the interface of SiNWs/Al_2_O_3_ is extremely challenging, and therefore, we estimated these parameters from those of c-Si/Al_2_O_3_, under the assumption that similar *D*_it_ and *Q*_ss_ can be expected. Figure 6 shows the values of *D*_it_ and *Q*_ss_ determined as a function of annealing time. In the absence of thermal annealing, the *Q*_ss_ at 400 and 500 °C was found to be 5.27 × 10^10^ and 1.0 × 10^10^ cm^−3^, respectively. We used different samples for the annealing experiments at 400 and 500 °C, and therefore, a small difference in the observed values of *Q*_ss_ was expected. The relationship between the surface recombination velocity and *Q*_ss_ was established as follows: the results showed that low values of *Q*_ss_ (below 10^11^ cm^−3^) did not reduce the surface recombination velocity [25]. As shown in Fig. 7a, *Q*_ss_ increased drastically after annealing. The Al_2_O_3_ layer fabricated on the Si generates negatively charged Al vacancies in the vicinity of the interface [23, 26]. The formation of Al vacancies represents one of the possible explanations for the origin of the negative fixed charge at the Al_2_O_3_/c-Si interface. The samples annealed at 400 and 500 °C exhibited almost identical *Q*_ss_. Therefore, the decrease in *τ*_eff_ cannot be explained by the values of *Q*_ss_. Figure 6b illustrates that *D*_it_ varied significantly with changes in the annealing temperature. For example, while the *D*_it_ at 400 °C was found to decrease with increasing time, the *D*_it_ at 500 °C exhibited the opposite trend. At the annealing time at 400 °C, the hydrogen from Al_2_O_3_ and FG can connect to the dangling bond. At annealing temperatures above 500 °C, on the other hand, the dangling bonds on the Si surface are generated as a result of bond disconnection between Si and H [27]. The results of our analysis showed that the value of *τ*_eff_ as a function of time was affected by the time-variable parameter *D*_it_. These results indicate that the SiNWs/Al_2_O_3_ annealing conditions significantly affect the interface quality.

## Conclusions

We investigated the influence of SiNW fabrication and annealing procedures on the effective lifetime of SiNW/Al_2_O_3_ samples. Complete crystallization of SiNWs is essential to obtain a high effective lifetime, in particular, because the a-Si phase remaining in the material can result in a decrease in the bulk lifetime of SiNWs. Here, fully crystallized thin-layer SiNWs were obtained by MAE of large-grain poly-Si. Through thermal annealing at 400 °C for 30 min in a forming gas, the effective lifetime of SiNWs/Al_2_O_3_ drastically improved from 2.86 to 15.9 μs. By comparing the effective lifetime of SiNWs/Al_2_O_3_ and c-Si/Al_2_O_3_ samples, we concluded that the thermal annealing conditions, especially temperature, significantly affect the state density at the SiNW/Al_2_O_3_ interface.

## Abbreviations

Al_2_O_3_: Aluminum oxide
MAE: Metal-assisted chemical etching
MWPCD: Microwave photoconductivity decay
SiNWs: Silicon nanowires
SPC: Solid phase crystallization
*τ*_eff_: Effective carrier lifetime
